# Arturo Casadevall Speaks on Curiosity, Resilience, and Scientific Integrity

**DOI:** 10.20411/pai.v10i2.921

**Published:** 2025-11-19

**Authors:** Robert A. Bonomo, Michael M. Lederman

**Affiliations:** 1 Medical Service, VA Northeast Ohio Health Care System, Louis Stokes Cleveland Department of Veterans Affairs Medical Center, Cleveland, Ohio; 2 Department of Biochemistry, Case Western Reserve University, Cleveland, Ohio; 3 Department of Medicine, Case Western Reserve University, Cleveland, Ohio; 4 Geriatric Research, Education and Clinical Center, Louis Stokes Cleveland Department of Veterans Affairs Medical Center, Cleveland, Ohio; 5 Departments of Pharmacology, Molecular Biology & Microbiology, and Proteomics & Bioinformatics, Case Western Reserve University, Cleveland, Ohio; 6 CWRU-Cleveland VAMC Center for Antimicrobial Resistance and Epidemiology (Case VA CARES), Cleveland, Ohio; 7 Case Western Reserve University School of Medicine, Cleveland, Ohio

**Keywords:** Biomedical Research, Infectious Diseases, Microbiology, HIV, AIDS, COVID-19, Scientific Integrity

## Abstract

In this interview, Arturo Casadevall, MD, PhD, shares insight into his childhood, what motivated him to go into biomedical research, the impact of the AIDS epidemic, and the lessons learned that he imparts to younger scientists.

## ROBERT A. BONOMO, MD

Welcome to our first *Pathogens and Immunity* interview with distinguished members of our discipline. It is a real pleasure today to bring to this forum Professor Arturo Casadevall. Dr. Casadevall is currently the Bloomberg Distinguished University Professor at Johns Hopkins University and he is also the chair of the Department of Molecular Biology and Immunology at Johns Hopkins University. Arturo is also known for his leadership as the founding editor of *mBio*, and as a former Deputy Editor of *The Journal of Clinical Investigation*.

A review of Dr. Arturo Casadevall’s curriculum vitae reveals accomplishments in many disciplines, including molecular biology and microbiology, fungal immunology and bacterial genetics, and in vaccine development. Arturo has also distinguished himself as being our conscience in medicine. He has called out many issues that have occurred in the areas of biodefense, biohazards, research integrity, and education. Arturo served leadership roles in the American Society for Microbiology and in our profession of Infectious Diseases. So it is with great pleasure that we invite Arturo here today.

Arturo, why don’t you tell us what your childhood was like.

## ARTURO CASADEVALL, MD, PHD

First, thank you, Robert, and thank you, Michael, for the honor of being interviewed. I was born and grew up the first 11years of my life in Cuba. It was such a difficult experience because my family was not happy with the government, and eventually even though they supported the government, and the revolution initially, by the late 60s they thought they had to leave, so we then left the country. Essentially the family split up and reunited in New York, and that’s where I spent most of my adult life.

## RAB

What were some of the influences that motivated you as a child to enter the field of medicine and biomedical research?

## AC

A very strong influence in my life was my grandfather, who was a surgeon. He was a very large presence in the first eight years of my life. In Cuba, we lived together as an extended family, but I didn’t know anything about research until I got to college.

First of all, we had the typical immigrant experience. I grew up in Elmhurst, New York, and the only place I could afford was the City University (CUNY), which was free at the time. We couldn’t afford anything, frankly. When I was in college, tuition was minimal by today’s standards, but I had to get a job. When I got to college, I spoke English very poorly, and by that time, I decided that the only way out of my situation was education. So, when I went to college, I realized that I could get good grades if I studied math and science, so I took primarily STEM courses.

Then I heard that there was something called a research elective, and I decided to go for an investigative career. I was a chemistry major and began to apply to graduate programs in chemistry and I was accepted. And then sometime around the end of my junior year, my father, who was a very influential person in my life, said to me, “So what are you going to do?” And I said, “I’m going to be a researcher.” He looked at me….. he wasn’t quite sure those jobs existed, but he knew another Cuban who knew about employment. This person advised my dad that I should go to medical school. So, my father confronted me in the kitchen and said to me, “You’re going to medical school.” And I said, “But I don’t have a plan to go to medical school.” He said, “You’re going to medical school. We’re going through a lot in this country. I want you to go to medical school. I want you to get a degree.”

I hadn’t taken pre-med courses, so I began to study for the MCAT on my own, basically completing the courses. Dad told me to go to medical school, so I went to medical school. I then figured out that there were things called MD/PhD programs, and they seemed to offer the ability to do research too, so I began to apply. The people at Queen’s College, a part of CUNY, said to me, “Look you’re wasting your time. They will never take anybody from the CUNY”

I was the first one they took. The interesting thing was when I told my dad about this, I said, “Dad, I got into an MD/PhD program.” He said, “You’re going to get a medical degree?” I said, “Yes. But I got better news for you. They’re going to pay tuition, and they gave me a stipend.” And he said, “What do you mean? What kind of racket is that? You mean they pay you to go to school?”

## MICHAEL M. LEDERMAN, MD

Was this always what you wanted to do, or if you didn’t go into medicine or biomedical research, what kind of career do you think you’d have had as your other choice?

## AC

In college, I had decided that I wanted a scientific career, so had I been left on my own devices, I may have ended up just going to graduate school. But with the “encouragement” of my dad, I was pushed into medicine, and then I found the opportunity to be able to do both. I love research. When I walked into the lab, the bug bit me. You know this idea that you are at the edge of knowledge, and then you go to work, and you find out new things that were not known before. That was so attractive.

## RAB

In what area did you do your PhD thesis? And then you decided to do your residency training at Bellevue University, correct?

## AC

I was always very attracted to the physical sciences, so I pursued my PhD in biophysical chemistry. I looked at light scattering and things like that, but I was already in medical school when AIDS was discovered in my second year. I then went to the wards in 1983 and 1984. You can imagine that this was the biggest thing that was happening, and I was enormously influenced by the tremendous suffering that I saw. And I then decided to go into Infectious Diseases, because this enormous calamity was happening. We didn’t know what was causing it, we didn’t know whether this was going to be contained. We didn’t know how the disease spread. So, once I graduated from medical school, I did a full residency in medicine and then a full fellowship in Infectious Diseases.

## MML

With the AIDS epidemic raging, what was it like?

## AC

First of all, many of the patients were my age, and many were younger. The majority of people who got admitted simply did not survive. It was a searing experience. There was nothing you could do for them. You could treat the infectious complications, you could treat their *Pneumocystis*, you could treat their Cryptococcal meningitis, the Toxoplasmosis. Before the end of my residency, AZT became available, and I was struck by the hope that came with that.

Think about it: We had a disease that came out of nowhere. It began to kill people who appeared to be healthy. There was nothing you could do, and then science, within a few years, delivers drugs, and these drugs only get better, such that by the 1990s, people no longer die from AIDS if they’re taking the (nucleoside analogue) drugs together with the protease inhibitors. This had an enormous influence on my thinking and on my optimism about what could be done, because I saw the power of science, and I saw that when it is applied, solutions emerge.

## RAB

In your early career as an independent investigator, what were some of the formative experiences or lessons you learned as a young investigator?

## AC

One important lesson was that it was okay to take risks, and that risks were really important. So, for example, there was no one in Einstein working with Cryptococcus, so by just working with Cryptococcus—I didn’t know it at the time, but I had created a research program on an organism, and once I began publishing, I became valuable in terms of being recruited internally when I finished my fellowship. The most important lesson, and one that I carry to this day, is to succeed in anything you have to persist and be determined. When I finished my fellowship, I could not get funded. I sent out dozens of applications. Eventually, the grants came, but that is a lesson, and that’s what I tell everybody: these jobs are hard. They have tremendous rewards, and that’s why we continue to do them, but success is just the ability to stick to it.

## RAB

Some of your work, in addition to the basic sciences, involves an analysis of integrity and honesty in medical research. How did you develop this interest in this area and how have you seen our field change as a result of your observations and insights?

## AC

In 2005, I was asked to be one of the editors of *Infection and Immunity*. A few years later, Ferric Fang became the editor-in-chief. When you’re an editor, you see the process of science from a different perspective. I became very upset with people rejecting work, calling it “descriptive.” It seemed to me there was a problem. All we know about the cosmos is descriptive, all we know about anthropology is descriptive, all we know about evolution is descriptive.

It just seemed to me that something was wrong somewhere, and I remember writing to Ferric and saying, “I’m tired of all these reviews putting down papers, calling them descriptive, and talking about mechanistic. I don’t think they have any idea what they’re talking about.” And then Ferric and I began to exchange emails and write essays, and I think we have published 60 or 70 essays on this. I think the first ones were Descriptive Science [1] and Mechanistic Science [2]. I began to realize that if the experts in the field were trashing work by calling it descriptive—when you could argue that all science is descriptive, because even if you find a new mechanism, you’re still describing it—that there were real problems in the way people were thinking.

## RAB

You said in one of your lectures that I attended that you wanted to bring back the “Philosophy into PhD,” please explain what you mean.

## AC

Some scientists, when they call work descriptive, want you to go into cause; they want you to do work to establish causality. Others are saying that they want you to go deeper, but they can’t often enunciate their criticism, so they call it descriptive. And, to me, that shows insufficient training in some of the fundamentals of science and philosophy: How do we know what we know?

## MML

You have been a successful editor, a successful researcher, a successful author, and a leader in terms of how people in our field think about science. How would you describe a paper that is a really good paper? Because some papers are not that interesting. A word that you could use that is maybe not the same as descriptive could be superficial, or not thoughtful. What does it take to be a good publication that you think should have a home in a good journal?

## AC

The most important papers change the way I think, but I don’t ever put down science. I don’t ever put down new work. You still have to go out there and do a lot of the analysis that people call descriptive. You need to know what’s there. That may not change your views or your thinking, but you’re still adding to the human knowledge base. Don’t denigrate it. On the other hand, an occasional paper, whether it is descriptive, describing a new thing, a new phenomenon, or whether it is mechanistic, establishing causality in ways you weren’t thinking about. You read it and you walk away thinking differently. Those papers to me are in a different category.

But I don’t put down work that is well done, even if it falls into more of the “regular work” category, because humanity can use any information that it can get, and some of these are pieces of a much larger puzzle. We need to encourage good work. Sometimes, if you’re just following good work that appears to be boring, it can lead you to a great discovery.

## MML

True enough; likely to be true, that should be enough, then?

## AC

es. And we need to do more to confirm the work. I think confirmatory work is very important. Don’t stop at, “it was done already.” Do it again. Let’s see if you get the same result. That’s really important.

## RAB

The topic of gender clearly has influence in authorship, and you’ve actually even addressed this in some of your papers, the issues of gender and authorship [3] and the issue of gender inequities and funding [4] in the field. You’ve thought a lot about this. Can you share with us?

## AC

I guess the provocative idea that we have is that scientists are really good at discriminating good science from bad science, but they are terrible when it comes to ranking good science. And the current system of funding requires scientists to rank things. A problem arises when science is ranked based on one’s liking one field more than another, or on one’s belief that the figures are nicer. These are not the issues that should go into deciding funding—and that’s, by the way, where gender bias comes in and ethnic bias comes in. The data show that women and underrepresented minorities don’t do as well in this system.

We published with Ferric a few years ago that, in fact, scientists cannot discriminate in the critical 20 percent range [5]. So, you have a situation in which scientists can’t discriminate, you ask them to do something they can’t do, and you have data that the current system hurts women and minorities. So, what I would say is: make two piles and then take the pile that is acceptable science and put it through a lottery. The current system is already a lottery, in that it is determined by chance who gets your grant, and in what order it gets reviewed in the morning or in the afternoon, and who or what else got reviewed before. This is not the way we should be funding science, but the lottery has one major advantage. The problem is that today we have a lottery, but it’s not random. At least if you have a lottery that has randomness, everyone who’s in the lottery has the same chance.

## RAB

You’ve worked with HIV. It clearly had a personal impact on you and influenced how you did research. Now you find yourself in the middle of the COVID-19 pandemic, and you have not shied away from this. You have been instrumental in shaping our thinking, shaping some clinical trials, and shaping how we look at the data from trials that have been both positive and even some trials that have failed. How were you able to pivot?

## AC

Know the history of your field. I trained in antibodies, and one of the things I did very early on was read a lot about antibody therapies, which were the mainstay of (antimicrobial) therapy up to 1940 when penicillin and sulfonamides replaced them, so I had that knowledge. In fact, I had written papers on these issues, and I knew that antibodies could be used for therapy, and I also knew that if you were going to use them, you better use them early. The evidence is in pneumococcal pneumonia. Antibodies don’t work after three days of symptoms. In meningococcal meningitis, evidence shows it doesn’t work after three days of symptoms. So, in 2020, it became clear to me, as an infectious disease person, this was not containable. Politicians and the health authorities were talking about vaccines, antivirals, and monoclonals. No one was talking about plasma. Plasma needs no development. All you need are recovered people.

The question is, “How do you get the word out?” Well, I wrote an op-ed, and I spent the entire month of February 2020 trying to get it published. I sent it to the *Wall Street Journal* and they published it in the February 27^th^ issue. If you read the op-ed [6], it basically says it works best if you use it early and, unfortunately, we spent a year in which the entire field had to be re-educated. The first use of convalescent plasma—a lot of it was for salvage. This is an antiviral, COVID-19 is a disease in which you have a viral phase, in which you can neutralize the virus with plasma. What gets you into the hospital is inflammation; what kills you is inflammation in the lungs. Antibody is not going to reverse an inflammatory process in the lung. So, it’s been a struggle in a way both trying to educate people and trying to get it to be used right. But, to me, I believe it has probably been one of the important things that I have done.

## RAB

What is the most important advice you can give young scientists embarking on an investigative career? You’ve been really successful. If you had to tell somebody “You need to do this.” How would you do it?

## AC

What I tell all the people who ask me is to become a generalist. In other words, you’re already a specialist, you’re already going through PhD training, you’re already specialized in infectious disease. Broaden yourself. You can do that on your own. The next most important thing to do is exercise your curiosity. Curiosity is like going to the gym. If you feed it, it drives itself. And they say, “Well how am I going to do that? I don’t have any time.” Every single day, try to learn a small fact about something else. And what happens is, if you learn one bit every day of the year, by the end of the year, you have 365 new bits, and you begin to make connections, and science is a lot more fun, you begin to see patterns. But you have to go outside your field. Don’t become somebody who says, “That’s not my field; I don’t have an opinion.” You’re a scientist. You should be a generalist; you should be ready to take on the world and new problems.

## RAB

Your last comment just before this one, I was going to quote you and say, “I’m preparing a lecture on something I know nothing about, and that uncomfortable feeling makes me grow.”

## AC

Because when you have to explain it to others, in areas that you don’t know, you’re going to process the information differently. I think we should spend more time in our uncomfortable zone.

## SUPPLEMENTARY VIDEO

Supplementary videos are available at the *Pathogens and Immunity* YouTube channel.
